# Transforming dementia diagnostics and care: developing a novel, cost‐effective technology to facilitate widespread and cost‐effective clinical implementation of neurofilament light and other biomarkers

**DOI:** 10.1002/alz70856_101930

**Published:** 2025-12-25

**Authors:** Dhamidhu Eratne, Jeff Smith, Kruti Patel, Christa Dang, Matthew JY Kang, Henrik Zetterberg, Kaj Blennow, Cherie Chiang, Dennis Velakoulis, The MiND Study Group

**Affiliations:** ^1^ Neuropsychiatry Centre, The Royal Melbourne Hospital, Parkville, VIC, Australia; ^2^ University of Melbourne, Parkville, VIC, Australia; ^3^ Walter and Eliza Hall Institute of Medical Research, Melbourne, VIC, Australia; ^4^ National Ageing Research Institute, Melbourne, VIC, Australia; ^5^ Department of Psychiatry and Neurochemistry, Institute of Neuroscience and Physiology, the Sahlgrenska Academy, University of Gothenburg, Molndal, Sweden; ^6^ Hong Kong Center for Neurodegenerative Diseases, Hong Kong, Science Park, China; ^7^ University College London, London, United Kingdom, London, United Kingdom; ^8^ Department of Psychiatry and Neurochemistry, University of Gothenburg, Gothenburg, Sweden; ^9^ Institute of Neuroscience and Physiology, Sahlgrenska Academy, University of Gothenburg, Gothenburg, Sweden; ^10^ The Royal Melbourne Hospital, Melbourne, VIC, Australia

## Abstract

**Background:**

Blood biomarkers such as neurofilament light chain (NfL) and phosphorylated tau (*p*‐tau) show great promise for improving the timely and accurate diagnosis of neurodegenerative dementias (ND) and distinguishing them from primary psychiatric disorders (PPD). However, current technologies, such as Quanterix Simoa, are limited by high costs and specialised infrastructure, hindering widespread implementation. A platform compatible with routine clinical pathology services could dramatically increase accessibility and adoption across diverse clinical settings.

**Method:**

We are developing a proximity ligation assay (PLA) for NfL. Early pilot data demonstrated successful detection of very low biomarker concentrations. Ongoing analyses utilise samples collected through routine clinical care from emergency, acute medical and psychiatric, memory clinic, and community settings, as part of The Markers in Neuropsychiatric Disorders Study (The MiND Study).

**Result:**

Preliminary data showed PLA detection correlates strongly with gold‐standard platforms like Quanterix Simoa (*R* = 0.86). Furthermore, PLA demonstrated high accuracy in distinguishing ND from PPD. Currently, analyses are underway in 300 participants across a broad spectrum of conditions, including Alzheimer's disease, other dementias, delirium, bipolar mania, depression, anorexia, and controls. As this study is underway and ongoing, the most up to date findings will be presented.

**Conclusion:**

A cost‐effective, high‐throughput PLA platform for NfL and other biomarkers, seamlessly integrated into routine clinical pathology workflows, could revolutionise diagnostics and research. This approach has the potential to improve access to timely and accurate diagnosis globally, leading to better outcomes for patients, families, and healthcare systems.

